# A case series of first rib resection patients assessed with a novel MRI protocol for neurogenic thoracic outlet syndrome

**DOI:** 10.1093/jscr/rjad672

**Published:** 2023-12-16

**Authors:** Phillip J Whiley, Rohit Tamhane, David T A Hardman

**Affiliations:** Department of General Surgery, The Canberra Hospital, Yamba Drive, Garran, Canberra, ACT 2605, Australia; The Australian National University Medical School, Yamba Drive, Garran ACT 2605, Australia; Canberra Imaging Group, 173 Strickland Crescent Deakin West, ACT 2600, Australia; Calvary John James Hospital, 173 Strickland Crescent, Deakin West, ACT 2600, Australia; University of Canberra, Centre for Research in Therapeutic Solutions 11 Kirinari St, Bruce ACT 2617, Australia

**Keywords:** thoracic outlet, MRI, first rib resection, outcomes

## Abstract

Selecting patients who will benefit from first rib resection for neurogenic thoracic outlet syndrome (nTOS) is made difficult by the variety of overlap symptoms with other musculoskeletal, neurogenic and psychological disease. A single diagnostic test is not available, and the diagnosis is typically made based on clinical findings and history. This case series assessed the utility of magnetic resonance imaging (MRI), with the patient’s arm placed in a symptom provoking position above the head, as a component of diagnosis nTOS and selection of patients to offer surgery. Outcomes from first rib resection were assessed using the guidelines of The Society for Vascular Surgery for Thoracic Outlet Syndrome. The cases demonstrate that the loss of perineural fat signal on MRI of the brachial plexus with the arm in the provocative position is a useful tool for assessing patients who would benefit from first rib resection for nTOS.

## Introduction

Thoracic outlet syndrome (TOS) is a neurovascular, chronic pain syndrome affecting nerves, arteries and veins either singularly or together. Depending on the structures crossing the first rib that are compressed, the pain syndrome will manifest as the neurogenic, venous or arterial subtype. The diagnosis of neurogenic TOS (nTOS) is clinical and while standardized assessment, imaging and neurophysical tests may assist in determining a site of compression [1–3], a single, diagnostic test is not available.

The neurogenic variant presents the most significant diagnostic challenge given the wide variety of diseases presenting with a similar pattern of symptoms. The paraesthesia and weakness associated with TOS are common features associated with cervical spine neuropathies, shoulder girdle injuries and arm neuropathies. In addition, pre-operative chronic psychiatric illness and socioeconomic factors are predictors of persistent functional loss after surgery for TOS [4]. This complexity in differential diagnoses emphasizes the need for precise history taking and patient selection and raises the need for a more specific diagnostic test.

First rib resection is considered a safe procedure [5]. Approximately half of patients who undergo surgery will be symptom free and 90% report improved symptoms in the long-term [6, 7]. For venous and arterial subtypes, >90% are free of symptoms after treatment [5] but predicting the patient who will benefit from surgery with the neurogenic TOS variant is difficult.

The evidence for outcomes from surgical intervention nTOS is generally positive however large-scale evaluation is limited [5, 8]. The diagnosis involves careful collection of the history, pattern of symptoms and examination findings to create a level of diagnostic suspicion [1, 9] however, the low specificity of clinical exam findings [10] indicate the need for a more specific investigation to clarify the diagnosis and provide objective support for the decision to provide surgical intervention.

A small number of studies have assessed the correlation between magnetic resonance imaging (MRI) performed with the arm in a neutral position then with the arm raised above the head replicating symptoms [11–13]. However, to date, MRI findings indicative of thoracic outlet brachial plexus compression in the provocative position has not been evaluated as a predictor of surgical outcome.

Predicting which patients will benefit from surgical intervention is difficult. This study presents a series of patients who have undergone a unique MRI protocol to capture brachial plexus compression with the arm raised above the head, provoking symptoms. The study presents the clinical course of this group of patients before and after surgery or physiotherapy.

## Case series

Twenty-three cases are presented from 19 patients (see Table 1, four patients had bilateral symptoms). Records of symptom history and physical examination findings were documented in accordance with the reporting standards of The Society for Vascular Surgery for Thoracic Outlet Syndrome [1]. Outcome measures included a functional assessment of the patient’s ability to carry out day-to-day tasks, limitations on work, social and recreational activities and an assessment of any hand and arm pain and paraesthesia.

**Table 1:** Series of patients with a brachial plexus MRI suggestive of nTOS between 2016 and 2020.

Case	Sex	Age at time of imaging	Duration of symptoms (months)	Symptoms			Examination findings				Suspicion of TOS	Improved with physio / pilates	Symptoms post-op over 12 months
				Paraesth-esia	Pain local	Pain peripheral	Point tender	ULTT or Elvey	Roos or EAST	Adson’s test			
1	F	18	60	✓	✓	✓	✓				med		Mild
2	F	19	18	✓	✓	✓	✓				med		None
3	M	20	12	✓		✓					high		Mild
4	M	20	6	✓	✓	✓	✓	✓	✓		high		None
5	F	21	60	✓		✓			✓	✓	high	✓	Mild
6	F	21	60	✓		✓			✓	✓	med	✓	None
7	M	21	12	✓		✓					high		Mild
8	M	21	12	✓		✓					med		Mild
9	M	23	24	✓					✓	✓	med		Severe
10	F	24	6	✓		✓					low		Nonea
11	F	26	60	✓		✓	✓	✓	✓		med	✓	Nonea
12	F	30	60	✓	✓	✓	✓	✓	✓		high	✓	Mild
13	F	33	6	✓	✓	✓	✓	✓	✓		med	✓	None
14	M	36	48	✓	✓	✓			✓	✓	med		Mild
15	M	38	24	✓		✓			✓		med	✓	Nonea
16	F	38	9	✓							med		Mild
17	M	43	100	✓							high	✓	Nonea
18	M	43	100		✓	✓	✓	✓	✓		high		Mild
19	M	43	18	✓	✓	✓	✓	✓	✓		med		Mild
20	F	43	36	✓							med		None
21	F	43	36	✓							med		None
22	M	46	12	✓	✓	✓		✓		✓	med		None
23	M	48	3	✓							med		None

Bilateral cases: 5/6, 7/8, 16/17, 19/20

^*^Non-operative management

## MRI protocol

Imaging was performed on a 3 T machine for all cases however, a 1.5 T machine was also used for four cases and the interpretation was comparable. The thoracic outlet MRI protocol was axial, coronal and sagittal T1, and fat saturated. T2 sequences with both arms in the neutral position were obtained with 340 mm × 300 mm field of view. In addition, sagittal T1 sequences were performed with both arms in neutral then elevated above the head with the field of view narrowed to 230 mm × 200 mm. T2 coronal stir sequence was also performed with the arms elevated to obtain a non-contrast angiogram of the thoracic outlet.

**Figure 1:**
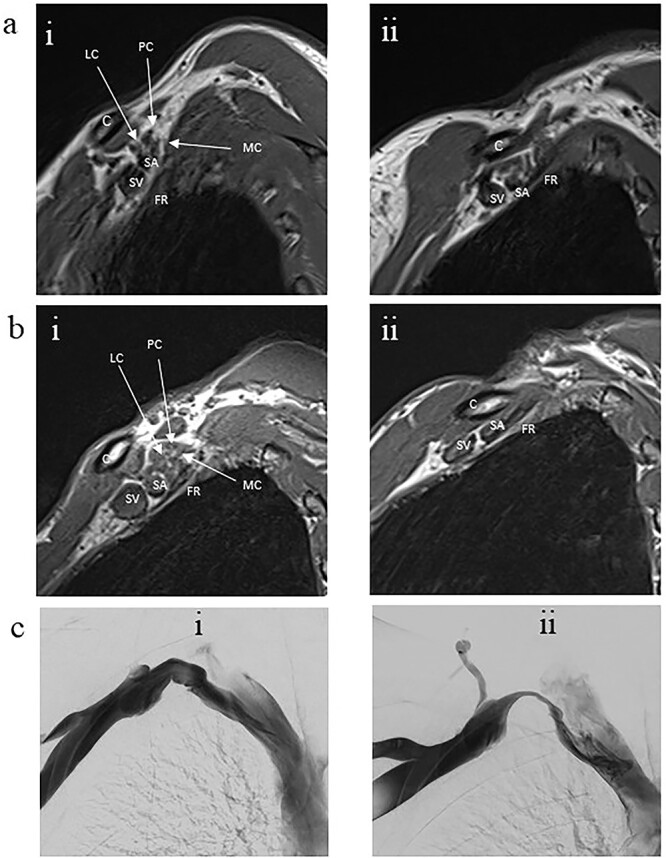
Panel a. Sagittal T1-weighted MRI of the costoclavicular space without imaging evidence of nTOS. Arm alongside the body (i) and arm elevated (ii). Maintenance of a fat plane surrounding the cords is seen with physiological narrowing of the space. Panel b. Sagittal T1-weighted MRI of the costoclavicular space with imaging evidence of nTOS. Arm alongside the body (i) and arm elevated (ii). Narrowing of the costoclavicular space and complete effacement of the fat indicating brachial plexus compression. Panel c. Venograms conducted with contrast injection in the antecubital vein while the arm is alongside (i) and in salute pose (ii) demonstrates tight stenosis of the subclavian vein and collateral flow. The MRI also showed effacement of the brachial plexus. The 36 year-old male subject had an excellent outcome after excision of the 1^st^ rib. C: Clavicle; FR: First rib, SV: Subclavian vein, SA: Subclavian artery, LC: Lateral cord, MC: Medial cord, PC: Posterior cord.

**Figure 2:**
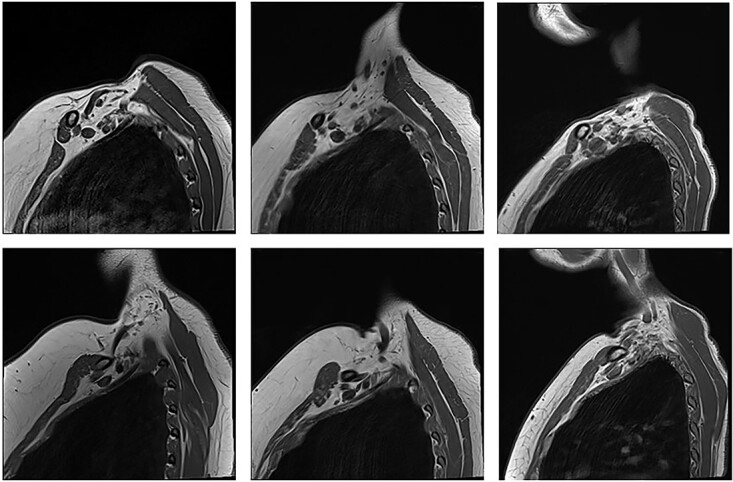
Unaffected patients. The top row are sequences with the arm in the neutral position. The bottom row is the corresponding patient with the arm raised.

**Figure 3:**
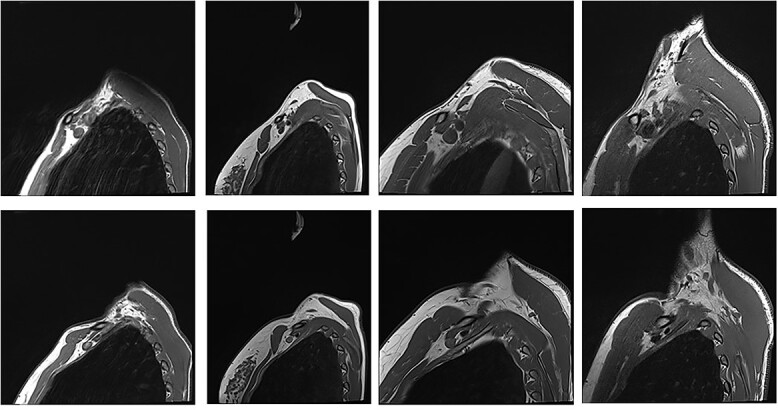
Patients with nTOS. The top rows are sequences with the arm in the neutral position. The bottom row is the corresponding patient with the arm raised.

With the arm elevated, there is narrowing of the costoclavicular space between the posterior border of the clavicle and anterior border of the first rib (Figs 1 and 3). In unaffected patients, there is a small amount of fat that persists around the cords of the brachial plexus with the arm elevated (Fig. 2). In our series, we considered a patient to be positive for nTOS if there is loss of the perineural fat signal demonstrating that the nerves are in direct contact with the posterior border of the clavicle and anterior border of the first rib (Fig. 1b).

We perform a 3D T2 coronal sampling perfection with application-optimized contrast using different flip angle evolution (SPACE) - short tau inversion recovery (STIR) sequence with a slice thickness of 2.5 mm with the arms elevated. This is a time-of-flight sequence that can demonstrate flow in the subclavian artery and vein. When interpreted in conjunction with the flow void of the subclavian artery on the sagittal T1 sequence, compression of the subclavian artery can be demonstrated.

## The cases

There were some noteworthy features associated with some individual cases:

Case 7: The clavicle was detached from the sternum after trauma, giving rise to nTOS.

Case 9: Did not respond well to surgery and there was no alternative explanation for his symptoms. This was the only case out of the 19 who underwent surgery and had a poor outcome.

Case 13: Initially, this patient was assessed by MRI without the provocative manoeuvre which showed no sign of brachial plexus compression. A repeat MRI with the new protocol subsequently showed evidence for cord irritation, the patient went on to have first-rib resection and had a positive outcome.

## Discussion

The treatment of nTOS with first rib resection typically results in positive clinical outcome. However, selecting patients who will benefit from surgery for the neurogenic type remains a challenge. This case series presents the clinical course, the findings from an evolved MRI protocol and treatment outcomes.

MRI of the thoracic outlet has been utilized to assess muscle thickness, first rib angle, interscalene angle and costoclavicular distance along with perineural fat disappearance in the provocative position [11]. MRI has also proven useful for detecting abnormal structures responsible for compression [14] but cannot be relied on to anticipate structural defects in every surgery [15]. To date, there is limited evidence of a correlation of MRI with provocative manoeuvres and outcomes from surgery. The combination of positive findings on MRI for extrinsic compression of the brachial plexus utilizing a protocol that positions the patient in the provocative position, combined with careful patient selection based on clinical and history features, strongly predicts those who will respond well to resection of the first rib. Patients with a history of chronic pain suspected complex regional pain syndrome and chronic pain medication use are difficult to treat when nTOS is a differential diagnosis. The findings in this study suggest that without a diagnostic MRI with the arm in the provocative position suggestive of brachial plexus compression, patients should not be selected for surgery.

Novel use of an MRI protocol of the brachial plexus demonstrating features of nTOS in this case series suggests that routine imaging with the arm raised is a key investigation preceding selection of appropriate patients to offer a first rib resection. The treatment outcomes presented in this series support the use of the MRI protocol to build confidence in the diagnosis of nTOS and support the selection of patients to offer first rib resection for nTOS.
